# Effect of a Multi-Carbohydrase and Phytase Complex on the Ileal and Total Tract Digestibility of Nutrients in Cannulated Growing Pigs

**DOI:** 10.3390/ani10081434

**Published:** 2020-08-17

**Authors:** Jia-Cheng Yang, Li Wang, Ya-Kuan Huang, Lei Zhang, Rui Ma, Si Gao, Chang-Min Hu, Jlali Maamer, Cozannet Pierre, Aurélie Preynat, Xin Gen Lei, Lv-Hui Sun

**Affiliations:** 1 Department of Animal Nutrition and Feed Science, College of Animal Science and Technology, Huazhong Agricultural University, Wuhan 430070, China; yangjiacheng@webmail.hzau.edu.cn (J.-C.Y.); huangyk@newhope.cn (Y.-K.H.); zhanglei6@webmail.hzau.edu.cn (L.Z.); marui6@webmail.hzau.edu.cn (R.M.); 2 Institute of Animal Science, Guangdong Academy of Agricultural Sciences, Guangzhou 510640, China; wangli1@gdaas.cn; 3 Demonstration Center of Hubei Province for Experimental Animal Science Education, Huazhong Agricultural University, Wuhan 430070, China; gaosi@mail.hzau.edu.cn; 4 College of Veterinary Medicine, Huazhong Agricultural University, Wuhan 430070, China; hcm@mail.hzau.edu.cn; 5 Adisseo France S.A.S., Center of Expertise in Research and Nutrition, 03600 Commentry, France; maamer.jlali@adisseo.com (J.M.); pierre.cozannet@adisseo.com (C.P.); aurelie.preynat@adisseo.com (A.P.); 6 Department of Animal Science, Cornell University, Ithaca, NY 14853, USA; XL20@cornell.edu

**Keywords:** multi-carbohydrase and phytase complex, fecal digestibility, ileal digestibility, nutrient, phytate, pig

## Abstract

**Simple Summary:**

It is well accepted that monogastric livestock lack phytase in their gastrointestinal tracts. Phytate (myo-inositol hexaphosphate, IP6) is the principal storage form of phosphorus (P) in many plant feeds, which can barely be used as a P source for animals. In addition, IP6 reduces the utilization of the proteins, amino acids, and minerals. Most of the IP6 is in the indigestible fibrous part of cereal grains, which also contain non-starch polysaccharides (NSP) that might also be beneficial for the availability of P, proteins, and amino acid. In this study, the multi-carbohydrase and phytase complex (MCPC) was tested to evaluate the effects on the ileal and total tract nutrients digestibility in growing pigs fed with low and high levels of phytate. The dietary supplementation of the MCPC improved the apparent ileal digestibility (AID) of P and calcium (Ca), along with the apparent total tract digestibility (ATTD) of crude fat, P, and Ca both in low and high phytate diet. Moreover, only a trend in enhanced protein digestibility was observed in the low phytate diet. In summary, the MCPC can be used in the diet of growing pigs which mainly promote the ADI and ATTD of P and Ca, which improve the efficiency of pig production.

**Abstract:**

The current study evaluated the influence of a multi-carbohydrase and phytase complex (MCPC) on the ileal and total tract digestibility of nutrients in growing pigs. A total of eight barrows (initial BW = 30.7 ± 1.1 kg) were surgically fitted with a T-cannula at the distal ileum and randomly allotted to four groups. The experiment was conducted according to a 4 × 4 Latin square design, each period lasting 10 days. Pigs were fed four experimental diets, which consisted of two basal diets (BD1, low phytate; BD2, high phytate) with or without MCPC containing at least 1800 U xylanase, 6600 U α-arabinofuranosidase, 1244 U β-glucanase, and 1000 U phytase per/kg corn–soybean meal with 15% corn distillers based diet. The high phytate diet reduced (*p* < 0.05) the apparent ileal digestibility (AID) of crude protein by 1.4% and the apparent total tract digestibility (ATTD) of organic matter, crude protein, and gross energy by 1.7, 2.3, and 1.9%, respectively, and tended to decrease (*p* = 0.10) the ATTD of Ca by 17.3%, relative to the low phytate diet. The dietary supplementation of the MCPC increased (*p* < 0.05) the AID of phosphorus (P) and calcium (Ca) by 34.2% and 31.1% for BD1 and 26.7% and 41.3% for BD2, respectively, and increased (*p* < 0.05) ATTD of crude fat, P, and Ca by 1.4%, 45.6%, and 9.6% for BD1 and 3.1%, 66.0%, and 52.7% for BD2, respectively. The MCPC supplementation did not significantly increase the AID and (or) ATTD of crude protein, organic matter, and starch. In conclusion, the dietary supplementation of the MCPC could improve the AID of P and Ca and the ATTD of crude fat, P, and Ca.

## 1. Introduction

The major storage form of phosphorus (P) in many plant feeds is phytate (myo-inositol hexaphosphate, IP_6_) [1]. Since monogastric livestock lack phytase in their gastrointestinal tracts, IP_6_ can barely be used as a P source for animals [2]. In addition, IP_6_ is capable of chelating minerals, including calcium (Ca), iron (Fe), zinc (Zn), manganese (Mn), and copper (Cu), and forming insoluble complexes, and any method enhancing P availability may increase the availability of these elements in feed as well [3]. In addition, IP_6_ can also bind to protein and reduce the utilization of the proteins and amino acids [4]. The addition of microbial phytase to pig’s diets has been widely used to breakdown the phytate-bound P and enhance P utilization from feed ingredients [5]. Most of the IP_6_ is in the indigestible fibrous part of cereal grains, which is also rich in anti-nutritional factors, such as non-starch polysaccharides (NSPs) [6]. NSPs are poorly digested by pigs in spite of significant hindgut fermentation, and thus can reduce the utilization of nutrients [7]. The dietary supplementation of NSPs degrading carbohydrases can reduce the NSPs-induced digesta viscosity and rupturing of NSP-containing cell wall, which makes the contents available for digestion [1,7]. Therefore, the combination of phytase and carbohydrase could be effective in improving the availability of P, Ca, proteins, and amino acid [1,7]. However, the results for phytase and carbohydrase combinations on nutrients utilization were inconsistent [8,9,10].

Therefore, the aim of this study reported herein was designed to evaluate the influence of supplementing a corn-soybean based diet with the newly developed multi-carbohydrase and phytase complex (MCPC) on the apparent ileal digestibility (AID) and apparent total tract digestibility (ATTD) of nutrients in growing pigs fed the diets with low and high phytate content.

## 2. Materials and Methods

### 2.1. Pigs, Diets, and Sample Collection

Our animal protocol was approved by the Institutional Animal Care and Use Committee of Huazhong Agricultural University, China (HZAUSW-2017-011). A total of 8 barrows (Duroc × Large White × Landrace; body weight at 30.7 ± 1.1 kg) were surgically fitted with a T-cannula at the distal ileum and randomly allotted into 4 groups. Pigs were housed in individual metabolic crate that allowed for the freedom of movement and allowed a 14-d recovery period. After the recovery, pigs were fed one of the 4 experimental diets and arranged in a 2 × 2 factorial design, which consisted of 2 basal diets (BD1, low phytate; BD2, high phytate; Table 1) to meet the nutritional requirements of NRC (2012), with or without MCPC (Rovabio Advance Phy, Adisseo France S.A.S., Antony, France) containing at least 1800 U xylanase, 6600 α-arabinofuranosidase, 1244 U β-glucanase, and 1000 U phytase per/kg diet. Rice bran is a rich source of phytate [11] and was used to create low/high phytate diets. The feed formulation software (Allix; A-Systems, Versailles, France) was used to estimate the nutrients values and formulate the diets. All diets contain 0.5% titanium dioxide (TiO_2_) as an indigestible marker and were fed as a mash form. The experiment was conducted on the basis of a double 4 × 4 Latin square design. The pigs were fed with a daily feed allowance at 4% body weight divided into 2 equal meals at times 08:00 h and 16:00 h and adjusted every week. Pigs had unlimited access to drinking water. Each experimental period lasted for 10 days, with the first 5 days serving as an adaptation period, followed by 3 days for feces collection and the last 2 days for ileal digesta sample collection. Feces were collected using plastic bags attached to the skin around the anus. Digesta samples were collected for 2 d via bags with diluted formic acid attached to the opened cannula barrel from 08:00 h to 20:00 h [12]. Digesta or feces samples were pooled for each pig within each experimental period. They were freeze-dried, ground and frozen at −20 °C for the subsequent chemical analyses.

### 2.2. Chemical Analyses

The organic matter, crude protein, gross energy, crude fat, starch, P, Ca, and ash in the BD were determined according to previous studies [13,14,15]. Briefly, the gross energy of samples was determined by an adiabatic oxygen bomb calorimeter according to procedures outlined by the Association of Official Analytical Chemists (AOAC, 1980) [16], and the concentration of starch was determined via the Ewers polarimetric method (EEC, 1972). In the AOAC (1990) [17] frame, the contents of Ca and P were determined by spectrophotometry; the content of crude protein was measured by Kjeldahl method (method 990-03); the content of crude fat was determined by diethyl ether extraction (method 920-39); the contents of ash and organic matter were determined after complete burned (methods 942-05 and 2001.12). The concentration of TiO_2_ was measured by the method, as previously described by Short et al. [18]. All the samples were analyzed in duplicate. The AID and ATTD of nutrients in the diet were calculated by the following equation as described before [12]:

AID or ATTD, % = 100 − [100 × (concentration of TiO_2_ in feed × concentration of component in feces or digesta)/(concentration of TiO_2_ in feces or digesta × concentration of component in feed)].

### 2.3. Statistical Analysis

Data were subjected to ANOVA using the General Linear Model using SAS (SAS Inst. Inc., Cary, NC, USA). The statistical model included the period, animal number, phytate level, MCPC and phytate × MCPC interaction. Tukey test was used in order to compare the means. Differences were considered significant at *p* < 0.05.

## 3. Results

The effects of phytate level and the MCPC on the AID of nutrients are presented in Table 2. Compared to the low phytate diet without the MCPC, the high phytate diet without the MCPC decreased (*p* < 0.05) the AID of crude protein by 1.4% units. Meanwhile, the high phytate diet with the MCPC decreased (*p* < 0.05) the AID of crude protein by 2.2% units, relative to the low phytate diet with the MCPC. Notably, the dietary supplementation of the MCPC increased the AID of P by 34.2% and 26.7% and the AID of Ca by 31.1% and 41.3% at low and high phytate levels, respectively. However, no significant interactions between phytate level and the MCPC was observed on the AID of these nutrients.

The effects of phytate level and the MCPC on the ATTD of nutrients and energy are presented in Table 3. The ATTD of organic matter, crude protein, crude fat and gross energy were significantly affected by dietary phytate content. High phytate diet without the MCPC decreased (*p* < 0.05) organic matter, crude protein and gross energy by 1.7, 2.3 and 1.9%, respectively, while it increased (*p* < 0.05) crude fat by 2.8% relative to the low phytate diet without the MCPC. The high phytate diet tended to decrease (*p* = 0.10) the ATTD of Ca by 17.3%, relative to low phytate diet in the −MCPC groups. Even though there was no significant interaction between the MCPC and phytate on all the measured nutrients, there might be a potential tendency of interaction between them. Specifically, the dietary supplementation of MCPC increased (*p* < 0.05) the ATTD of crude fat by 1.4 and 3.1%, the ATTD of P by 45.6 and 66.0%, and the ATTD of Ca by 9.6 and 52.7% at low and high phytate levels, respectively. However, no treatment effect was observed on starch ATTD.

## 4. Discussion

The current study showed that the ATTD of organic matter, crude protein, gross energy, and Ca, and the AID of crude protein, were significantly reduced by the dietary phytate content in growing pigs, which is in agreement with the observations made by Woyengo et al. [19] and Kahindi et al. [20]. Phytate can bind to starch, protein and glucose and may also reduce the enzyme activities of both amylase and protease in the small intestine, hence reducing the digestibility and utilization of nutrients [3,4,21,22,23]. It is generally accepted that by breaking down phytate, phytase limits the insoluble complex formation between phytate and nutrients, thus improving nutrient digestibility [5,24,25]. Interestingly, the current study showed that the dietary supplementation of enzymes rich in phytase and multi-carbohydrase increased the AID of P and Ca, as well as the ATTD of crude fat, P, and Ca in both low and high phytate diets. This could be explained by the enzyme degradation of IP_6_ and polysaccharides in the cell walls in cereals and storage cell contents in protein meals and reduction in digesta viscosity [5,7]. These results were the same as previous studies, which showed that the dietary supplementation of either phytase, NSP degrading enzymes, or both improved the AID and ATTD of crude fat, P, and/or Ca in swine [8,26,27,28,29]. Moreover, the current study potentially revealed that the supplementation of phytase plus multi-carbohydrase rich in xylanase, α-arabinofuranosidase and β-glucanase increased the ATTD of P (66.0%) and Ca (52.7%) in a higher phytate diet than the dietary supplementation of phytase at 1000 FTU/kg only, which increased the ATTD of P by 32.2% and Ca by 15.5% [30]. These outcomes are consistent with a previous study that showed the dietary supplementation of phytase and xylanase together displayed better results of the AID and ATTD of P and Ca than dietary supplementation of xylanase alone [1]. These findings could be explained by a complementary effect between multi-carbohydrase and phytase on the digestibility of both minerals. This is due to that the multi-carbohydrase degrades NSPs on the aleurone layer where phytate is found, which makes phytase more readily accessible to phytate resulting in a more pronounced effect of phytase in increasing P and other nutrients digestibility [1,6,7].

Nevertheless, several inconsistent scenarios were observed in the present study. The current study showed that dietary supplementation of enzymes did not affect the AID and ATTD of crude protein, as presented by Lindberg et al. [1] and Woyengo et al. [8]. Additionally, the ATTD of starch was not affected either by the phytate level or by the MCPC supplementation. However, some other studies showed that the dietary supplementation of either phytase or NSP-degrading carbohydrase alone or in combination can improve the digestibility of crude protein and starch [31,32,33]. These discrepancies could also be related to the variations in the diet compositions, doses and types of enzymes, and ages of pigs. Strikingly, dietary high levels of phytate increased the crude fat digestibility, which might be explained by the high fat content with unsaturated fatty acids from rice bran and higher soybean oil in the high phytate diet relative to the low phytate diet in this study [34,35]. Moreover, in this study, the digestibility of nutrients was not affected by the interaction between phytic P level and MCPC. These outcomes are not in agreement with previous studies [31,33], which could be due to the different experimental conditions, including diet compositions, doses of enzymes, and ages of pigs.

## 5. Conclusions

In conclusion, the present study revealed that the dietary supplementation of an enzymatic cocktail with phytase and multi-carbohydrase improved the ileal and total tract digestibility of P, Ca, and crude fat in growing pigs and could be practically applied as a promising enzymes product to reduce the negative effects of phytate in nutrient availability.

## Figures and Tables

**Table 1:** Ingredients and nutritional compositions of basal diets ^1^.

Ingredients	Percentage (%)		Calculated Nutrients (% DM)	Content ^4^	
	BD1	BD2		BD1	BD2
Corn	58.00	50.61	GE ^5^, MJ/kg	16.70	17.40
Corn distillers	15.00	15.00	NE, MJ/kg	9.80	9.80
Rice bran	2.53	15.00	ME, MJ/kg	12.67	12.77
Soybean meal	13.25	6.00	Crude protein ^5^	18.48	16.85
Rapeseed meal	7.00	7.00	Crude fat ^5^	5.44	7.73
Soybean oil	1.09	2.94	Ash ^5^	5.14	5.23
DL-methionine	0.00	0.05	Ca ^5^	0.70	0.68
L-lysine	0.42	0.59	Total P ^5^	0.52	0.61
L-threonine	0.03	0.11	Phytic P	0.31	0.46
L-tryptophan	0.02	0.04	SID Lysine	0.95	0.95
CaCO_3_	1.09	1.10	SID Methionine	0.28	0.31
CaPO_3_	0.20	0.19	SID Methionine+cysteine	0.52	0.52
Salt	0.37	0.37	Starch ^5^	44.75	45.61
Titanium oxide	0.50	0.50	Soluble arabinoxylan	0.77	0.66
Mineral premix ^2^	0.25	0.25	Insoluble arabinoxylan	5.60	5.01
Vitamin premix ^3^	0.25	0.25	Total arabinoxylan	6.37	5.67
Total	100	100			

^1^ BD = based diet; BD1 = low phytate; BD2 = high phytate; Ca = calcium; P = phosphorus; GE = gross energy; ME = metabolic energy; NE = net energy; SID = standardized ileal digestibilities. ^2^ Mineral premix provided/kg diet: cholecalciferol 2500 IU; retinyl acetate, 10,000 IU; dl-α-tocopheryl acetate, thiamin, 2.0 mg; 50 IU; menadione, 5.0 mg; riboflavin, 5.0 mg; pyridoxine, 10.0 mg; pantothenic acid, 12.0 mg; niacin, 30.0 mg; folic acid, 1.5 mg; biotin, 0.2 mg; choline chloride 1500 mg; cyanocobalamin 0.05 mg. ^3^ Vitamin premix provided/kg diet: iron, 100 mg; zinc, 100 mg; copper, 20 mg; selenium, 0.3 mg; manganese, 25 mg; iodine, 0.3 mg. ^4^ Calculated values. ^5^ Measured values.

**Table 2:** Effects of phytate level and enzymes on the AID of nutrients ^1^.

	Low Phytate		High Phytate			*p*-Value		
	−MCPC	+MCPC	−MCPC	+MCPC	SEM	Phytate	MCPC	Phytate × MCPC
Organic matter	82.59	82.62	82.77	83.38	0.22	0.14	0.31	0.35
Crude protein	82.35	83.69	81.23	81.87	0.40	0.04	0.14	0.60
P	47.24	63.40	50.60	64.09	1.56	0.17	<0.01	0.36
Ca	47.60	62.40	43.67	61.69	1.87	0.33	<0.01	0.51

^1^ Data (n = 32) were subject to variance analysis with fixed effect phytate (n = 2), MCPC (n = 2) and interaction (n = 4). AID = apparent ileal digestibility; MCPC = multi-carbohydrase and phytase complex; −, diet without added MCPC; +, diet with added MCPC; P = phosphorus; Ca = calcium.

**Table 3:** Effects of phytate level and enzymes on the ATTD of nutrients ^1^.

	Low Phytate		High Phytate			*p*-Value		
	−MCPC	+MCPC	−MCPC	+MCPC	SEM	Phytate	MCPC	Phytate × MCPC
Organic matter	85.82	85.49	84.33	84.57	0.32	<0.01	0.89	0.42
Starch	91.85	91.56	91.75	91.97	0.20	0.72	0.93	0.55
Crude protein	81.52	83.28	79.63	79.92	0.62	0.001	0.15	0.30
Crude fat	79.11	80.22	81.29	83.82	0.70	<0.001	<0.01	0.22
Gross energy	83.19	82.56	81.64	82.05	0.40	0.03	0.81	0.24
P	32.67	47.57	27.55	45.72	2.11	0.20	<0.01	0.54
Ca	49.59	54.34	40.99	62.60	2.27	0.10	<0.01	0.28

^1^ Data (n = 32) were subject to variance analysis with fixed effect phytate (n = 2), MCPC (n = 2) and interaction (n = 4). ATTD = apparent total tract digestibility; MCPC = multi-carbohydrase and phytase complex; −, diet without added MCPC; +, diet with added MCPC; P = phosphorus; Ca = calcium.
